# HSPG2 overexpression independently predicts poor survival in patients with acute myeloid leukemia

**DOI:** 10.1038/s41419-020-2694-7

**Published:** 2020-06-30

**Authors:** Xiaojia Zhou, Simin Liang, Qian Zhan, Li Yang, Jianxiang Chi, Li Wang

**Affiliations:** 1 Department of Hematology, The First Affiliated Hospital of Chongqing Medical University, Chongqing, China; 2 The Center for Clinical Molecular Medical detection, The First Affiliated Hospital of Chongqing Medical University, Chongqing, China; 3 Center for the Study of Hematological Malignancies, Karaiskakio Foundation, 2032 Nicosia, Cyprus

**Keywords:** Tumour biomarkers, Prognostic markers

## Abstract

Heparan sulfate proteoglycan 2 (HSPG2), also known as perlecan, is a large multi-domain extracellular matrix proteoglycan, which contributes to the invasion, metastasis and angiogenesis of solid tumor. However, very little is known about the effect of HSPG2 on acute myeloid leukemia (AML). This study aims to investigate the prognostic value of the HSPG2 gene in terms of overall survival and leukemia-free survival in patients with AML. Bone marrow mononuclear cells (BMMCs) from 4 AML patients and 3 healthy controls were processed for RNA-Sequencing (RNA-seq). The mRNA expression level of HSPG2 in BMMCs and CD34^+^ hematopoietic stem/progenitor cells (HSPC) obtained from enrolled participants and human leukemic cell lines was detected by RT-qPCR. Then the correlations between the expression of HSPG2 and a variety of important clinical parameters, such as median white blood cell (WBC) count and bone marrow (BM) blasts, were further analyzed. The expression level of HSPG2 was significantly upregulated in AML patients at the time of diagnosis, downregulated after complete remission and then elevated again at relapse. Moreover, HSPG2 expression was associated with median WBC count (*P* < 0.001), median hemoglobin (*P* = 0.02), median platelet count (*P* = 0.001), and BM blasts (*P* < 0.001) in AML patients. Patients with high HSPG2 expression had both worse overall survival (OS) (*P* = 0.001) and poorer leukemia-free survival (LFS) (*P* = 0.047). In the multivariate analysis model, HSPG2 was identified as an independent prognostic biomarker of AML. Taken together, these results indicate that HSPG2 overexpression was associated with poor prognosis in AML patients, and may be a prognostic biomarker and therapeutic target of AML.

## Introduction

Acute myeloid leukemia (AML) is a group of malignant myelopoietic stem/progenitor cell diseases, characterized by abnormal proliferation of primitive and immature myeloid cells in bone marrow and peripheral blood^1,2^. Genetic abnormalities, including chromosomal abnormalities, gene mutations and gene expression abnormalities, involved in the pathogenesis of AML. For example, mutations in NPM1, FLT3-ITD, and c-KIT, as well as aberrant expression of BCL-2, MN1, WT1, and MDR1 provide some clues for evaluating the prognosis of AML patients^3–5^. Although most AML patients achieve complete remission (CR) after chemotherapy and allogeneic hematopoietic stem cell transplantation (allo-HSCT), the 5-year overall survival rate of AML patients is still poor^6^. Drug resistance and relapse are the two main reasons that affect the survival of patients after transplantation. Therefore, it is of great clinical significance to identify new biomarkers for predicting prognosis and optimizing individual treatment strategies.

HSPG2 (Perlecan), a heparan sulfate proteoglycan, is a protein encoded by HSPG2 gene (maps to 1p36.12 in the human genome)^7–9^. 1p36 is a Carcinoma Prostate Brain (CAPB) locus that hosts a series of oncogenes for prostate cancer and the brain tumor^10^. Functionally, HSPG2 is one of the major components of the vascular extracellular matrix and basement membranes^11^. Previous studies have showed that high expression of HSPG2 might be an indicator of prostate cancer grade, invasion potential and distant metastasis^10,12^. Besides, it is reported that the overexpression of HSPG2 predicts poor survival in patients with oligoastrocytoma and oligodendroglioma^13,14^. An in vitro study found that HSPG2 is actively synthesized by bone marrow derived cells and the abnormal expression of HSPG2 might play a vital role in hematopoietic cell differentiation^15^. However, the role of HSPG2 remains unknown in AML.

In this study, RNA-sequencing (RNA-seq) and differential expression analysis found that the overexpression of HSPG2 was higher in AML patients significantly than in healthy controls. The role of HSPG2 remains unknown in AML, although overexpression of HSPG2 has been well studied in other diseases. Our research aimed to detect the expression of HSPG2 in AML patients and assess the influence of HSPG2 expression on the clinical characteristics and prognosis, which might contribute to explore new biomarker and therapeutic target of AML.

## Materials and methods

### Patients and clinical characteristics

Bone marrow specimens and clinical data were obtained from patients who were diagnosed with AML between 2014 and 2017. In total 151 AML patients and 11 healthy controls were enrolled in this study. The use of bone marrow specimens was approved by the Ethics Committee of the First Affiliated Hospital of Chongqing Medical University (2020–264). Written informed consent was obtained from all participants. Bone marrow mononuclear cells (BMMCs) from the participants were separated by density-gradient centrifugation with Lymphocyte Separation Medium (TBD, China, LTS1077). The main clinical and laboratory features of the patients were presented in Table 2.

### CD34+ cell sorting

Normal bone marrow CD34^+^ cells and AML bone marrow CD34^+^ cells were isolated from BMMCs by using EasySep^TM^ Human CD34 Positive Selection Kit II (Stemcell Technologies, Canada). Briefly, 1 × 10^8^ cells were incubated with 20 μL Selection Cocktail for 30 min at room temperature. Then, the cells were incubated in a magnet with 15 μL RapidSphere^TM^ for 5 min at room temperature. After pouring off the supernatant, cells were resuspended in 1 mL of PBS to pick up the magnet coupled with CD34^+^ cells in five times in order to yield a high purity (>95%). Finally, the purity of collected CD34^+^ cells was estimated by flow cytometry (FACSCalibur analyzer, BD Biosciences, USA).

### Cell lines and cell culture

The SKM-1, an AML-MDS cell line, was provided by Professor Jianfeng Zhou working in Tongji Medical College of Huazhong University of Science and Technology (Wuhan, China). Chronic myeloid leukemia cell line K562 was provided by Chongqing Key Laboratory of Translational Medicine in Major Metabolic Diseases. The HS-5, a human bone marrow stromal cell line, was provided by Department Molecular Diagnostic Center for Clinical Medicine, the First Affiliated Hospital of Chongqing Medical University (Chongqing, China). Human peripheral blood leukemia T cells line Jurkat was provided by Children’s Hospital of Chongqing Medical University (Chongqing, China).

HS-5 cells were cultured in DMEM medium (Gibco/Thermo Fisher Scientific, Waltham, MA, USA) with 10% fetal bovine serum (FBS, PAN seratech, Germany) and 1% penicillin/streptomycin (PS, Beyotime, China). Three human leukemia cell lines (SKM-1, K562, and Jurkat) were cultured in RPMI 1640 medium (Gibco/Thermo Fisher Scientific, USA) supplemented with 10% FBS (PAN seratech, Germany) and 1% PS (Beyotime, China). The cells were cultured in a humid atmosphere at 37 °C with 5% CO_2_.

### RNA isolation and quantitative real-time polymerase chain reaction (RT-qPCR)

Total RNA was isolated from each sample using trizol reagent (Beyotime, China). Complementary DNA (cDNA) was synthesized from total RNA with reverse transcription kit (Takara, Japan) according to the manufacturer’s instructions. RT-qPCR was performed using CFX96 Real-Time PCR Detection System (BIO RAD, USA). The total reaction volume was 10 µL and was prepared as follows: 5 µL of TB Green (Takara, Japan), 0.4 µL of each primer (10 µmol/L), 1 µL of cDNA template (0.5 ng/µL), and 3.2 µL of ddH2O. The cycling conditions were as follows: 95 °C for 30 s, followed by 40 cycles at 95 °C for 5 s and 60 °C for 30 s. Transcript levels were normalized vs. β-actin expression. The gene expression was calculated using the formula 2^−ΔΔCt^.

The primers sequences were as follows:

HSPG2 Forward 5’-GACATCGCCATGGATACCAC-3’

Reverse 5’-CAGGACAAGCCAGAATAGCC-3’

β-actin Forward 5’-CATTGCCGACAGGATGCAG-3’

Reverse 5’-CGGAGTACTTGCGCTCAGGA-3’.

### Western blot

Total protein was harvested from each sample using RIPA lysis buffer (Beyotime, China) supplemented with 1 µM phenylmethanesulfonyl fluoride (PMSF, Beyotime, China). Then, the extract was digested with 0.01 units/ml heparinase III (Sigma, USA, H8891) at 37 °C for 3 h. Thirty microgram of protein was separated by sodium dodecyl sulfate-polyacrylamide gel electrophoresis (SDS-PAGE, GenScript, USA) and transferred onto polyvinylidene fluoride membranes (PVDF, Millipore, USA). Subsequently, membranes were blocked with 5% non-fat milk for 2 h at room temperature, followed by incubation with primary antibodies at 4 °C overnight. The primary antibodies used in this study were anti-HSPG2 (SantaCruz, USA, sc-33707) used at 1:1000 and anti-β-actin (Bioss, China, bs-0061R) used at 1:5000 diluted in primary antibody dilution buffer (Beyotime, China, P0256). The membranes were washed and exposed to corresponding horseradish peroxidase (HRP)-conjugated goat anti-rat (1:5000, Mengbio, China, MS002A) or goat anti-rabbit (1:1000, Beyotime, China, A0208) secondary antibodies diluted in TBST buffer for 1 h at room temperature. Finally, the protein bands were visualized with an enhanced chemiluminescence (ECL) kit (Advansta, USA, K-12045-D10), and the band intensity was analyzed using Vilber Fusion software (Fusion FX5 Spectra, France).

### RNA-seq

Paired-end libraries were synthesized by using the TruSeq™ RNA Sample Preparation Kit (Illumina, USA) following TruSeq™ RNA Sample Preparation Guide. Briefly, the poly-A containing mRNA molecules were purified using poly-T oligo-attached magnetic beads. Following purification, the mRNA is fragmented into small pieces. The cleaved RNA fragments are copied into first strand cDNA. This is followed by second strand cDNA synthesis using DNA Polymerase I and RNase H. These cDNA fragments then go through an end repair process, the addition of a single ‘A’ base, and then ligation of the adapters. The products are then purified and enriched with PCR to create the final cDNA library. Cluster was generated by cBot with the library diluted to 10 pM and then was sequenced on the Illumina NovaSeq 6000 (Illumina, USA). The library construction and sequencing were performed at Shanghai Sinomics Corporation, followed by data analysis they performed. The criteria for differential genes was set up as fold change >2 or <−2, *P*-value <0.05, and FDR < 0.05.

### Statistical analysis

Data were analyzed using SPSS 22.0 (SPSS Inc., USA) and GraphPad Prism 5.01 (GraphPad Software Inc., USA). Complete remission (CR) was defined as normalization of peripheral blood and bone marrow (<5% blasts in the bone marrow; absence of extramedullary disease; absolute neutrophil count >1.0 × 10^9^/L; platelet count >100 × 10^9^/L), and absence of clinical symptoms^16^. Overall survival (OS) was measured from diagnosis to last follow-up or death from any cause. Leukemia-free survival (LFS) was calculated from the day that CR was established until either relapse or death without relapse^17^. Mann–Whitney’s U test and Pearson Chi-square analysis/Fisher exact test were applied to compare the difference of continuous variables and categorical variables between two groups, respectively. The ROC curve and area under the ROC curve (AUC) were to assess the discriminative capacity of HSPG2 expression among patients and controls. The univariate Kaplan–Meier (KM) method and the multivariate Cox proportional hazard regression model were used to determine the survival curve and independent risk factors of AML patients. Spearman was used to analyze the correlation between the expression level of HSPG2 and clinicopathological factors. For all tests, a *P*-value < 0.05 was considered as statistically significant. All experiments were performed in triplicate.

## Results

### The expression of HSPG2 in participants and cell lines

**Fig. 1:**
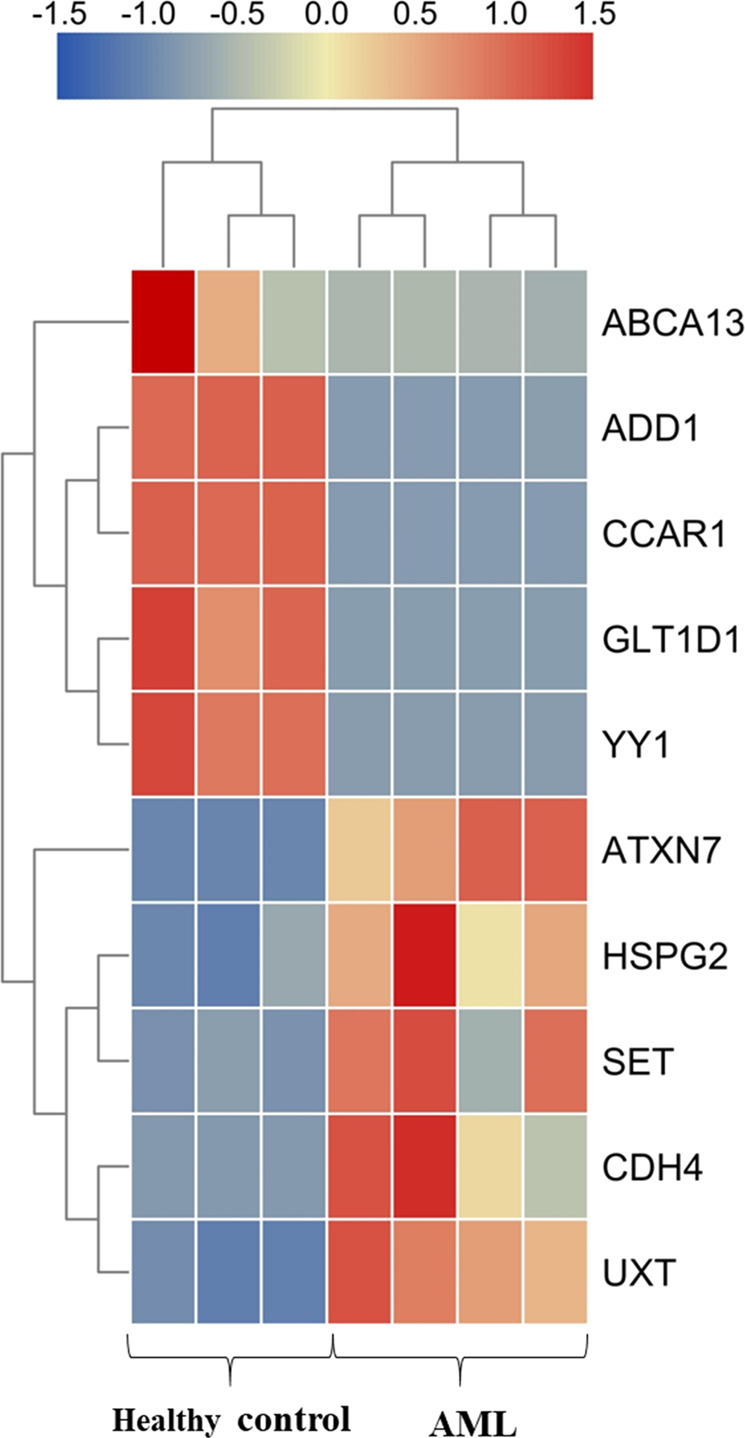
Hierarchical clustering of 10 differentially expressed genes in AML patients and healthy controls. Upregulated genes are shown in red and downregulated genes are shown in blue.

**Fig. 2:**
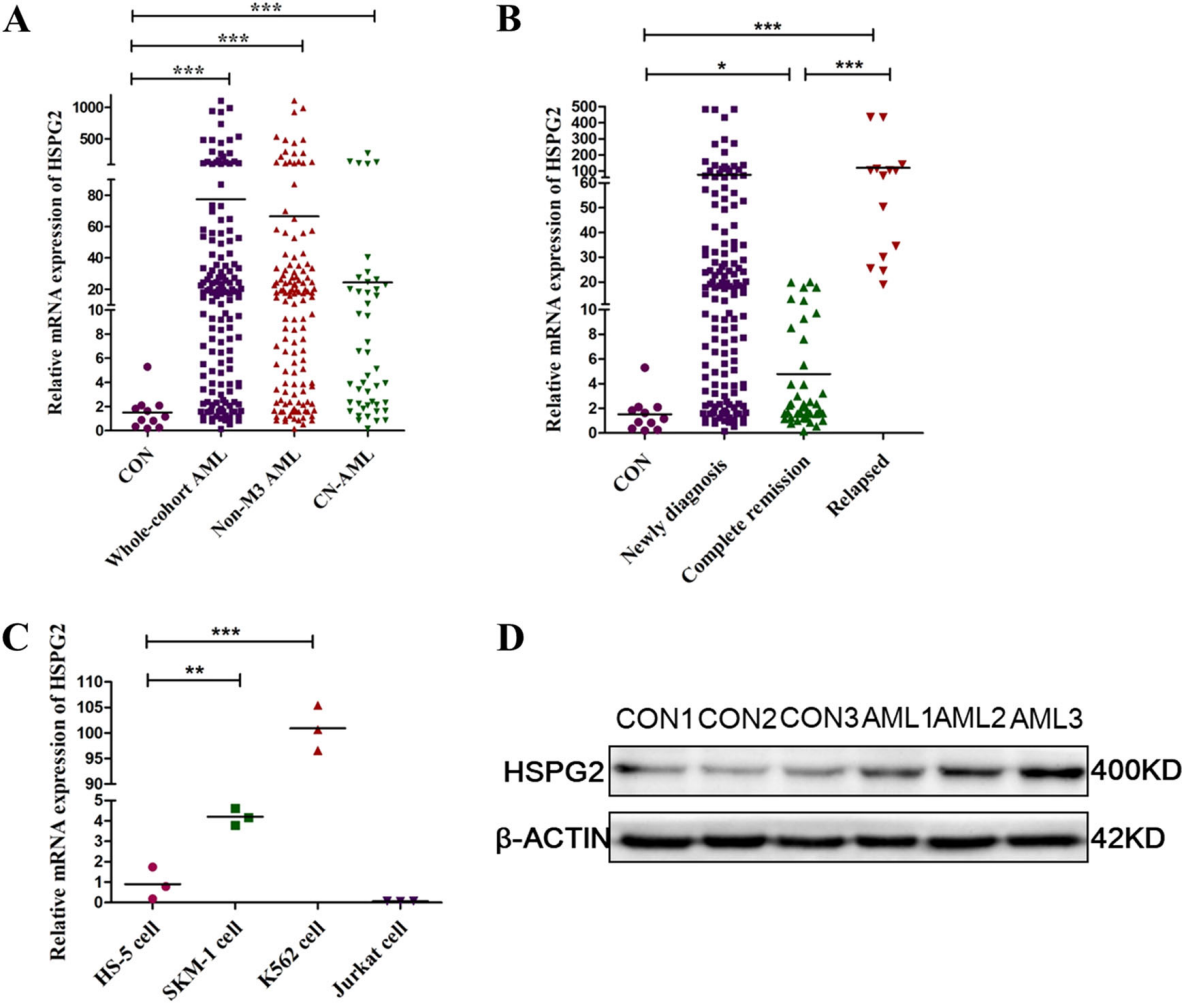
Relative expression level of HSPG2 was normalized to β-actin. **a** in healthy controls and AML patients by RT-qPCR. **b** in different stage of AML by RT-qPCR. **c** in cell lines by RT-qPCR. **d** Western blot analysis of HSPG2 performed on healthy controls and on AML patients. β-actin was used as a normalizer. AML acute myeloid leukemia, Non-M3 AML non M3 acute myeloid leukemia, CN-AML cytogenetically normal acute myeloid leukemia, CON control. All the data are presented as the means ± SDs and analyzed by GraphPad Prism 5.01, *P < 0.05, **P < 0.01, and ***P < 0.001.

The results of RNA-seq analysis indicated HSPG2 significantly upregulation in AML patients compared with healthy controls (Fig. 1 and Table 1). As shown in the results of RT-qPCR, the expression of HSPG2 was significantly upregulated in AML patients (Fig. 2a, *P* < 0.001), and human myeloid leukemia cell lines (SKM-1 cells and K562 cells) (Fig. 2c, *P* < 0.001), but not in human T cell acute lymphoblastic leukemia cell line (Jurkat cells) (Fig. 2c, *P* > 0.05). The mRNA expression of HSPG2 in CD34^+^/CD34^−^ cells from AML patients was higher than that in CD34^+^/CD34^−^ cells from healthy controls (Fig. S1). Furthermore, the protein expression of HSPG2 was higher in AML patients than that in healthy controls (Fig. 2d).

**Table1:** Ten differentially expressed genes between AML patients and healthy controls.

Gene id	Gene name	log2FC	Up/down
ENSG00000163635	ATXN7	9.85	Up
ENSG00000179242	CDH4	Inf	Up
ENSG00000142798	HSPG2	3.37	Up
ENSG00000126756	UXT	7.09	Up
ENSG00000119335	SET	7.90	Up
ENSG00000151948	GLT1D1	#NAME?	Down
ENSG00000087274	ADD1	−9.58	Down
ENSG00000179869	ABCA13	−6.99	Down
ENSG00000100811	YY1	#NAME?	Down
ENSG00000060339	CCAR1	#NAME?	Down

Inf represents a gene which was not detected in healthy controls group; #NAME? represents a gene which was not detected in AML patients group.

### Discriminative capacity of HSPG2 expression

**Fig. 3:**
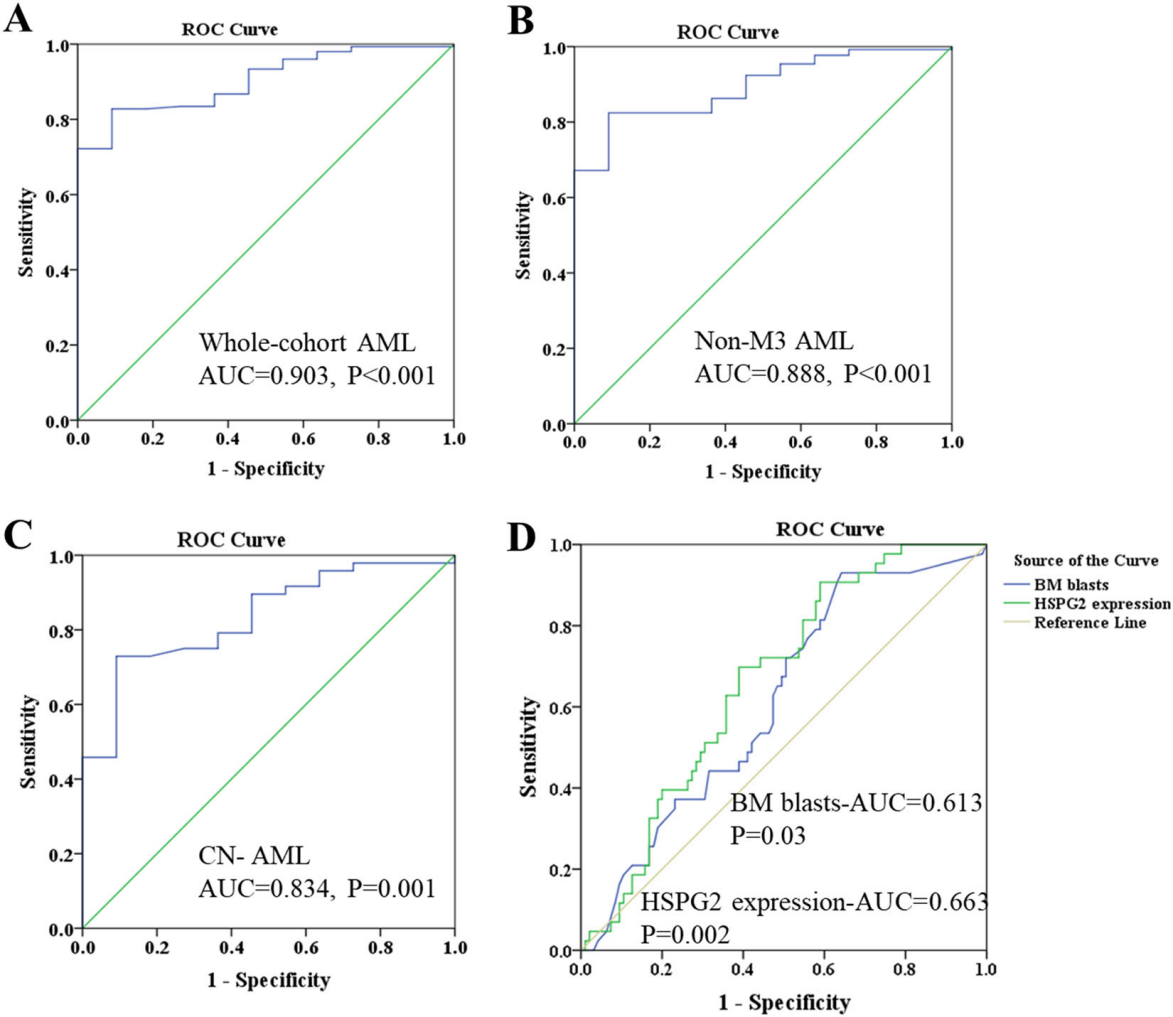
ROC curve analysis of HSPG2 expression for discriminating acute myeloid leukemia patients from controls. **a** in whole‐cohort AML. **b** in non‐M3 AML. **c** in CN‐AML. **d** The ROC curves of BM blasts and HSPG2 expression for predicting death within 2 years in whole-cohort AML. AML acute myeloid leukemia, AUC area under the ROC curve, Non-M3 AML non M3 acute myeloid leukemia, CN-AML cytogenetically normal acute myeloid leukemia, BM bone marrow.

ROC curve was used to examine the diagnostic value of HSPG2 in AML patients compared with healthy controls and found the AUC value to be 0.903 in whole-cohort AML (95% confidence interval (CI): 0.842–0.965, *P* < 0.001; Fig. 3a) with a sensitivity of 88.0% and a specificity of 74.1%, which indicated that HSPG2 could be a potential biomarker for distinguishing AML patients from healthy controls. Similarly, distinguishing capacity of HSPG2 expression was also shown in non-M3 AML and cytogenetically normal AML patients (AUC = 0.888, 95% CI: 0.814–0.963, *P* < 0.001; AUC = 0.834, 95% CI: 0.719–0.949, *P* = 0.001; respectively; Fig. 3b, c). Furthermore, to determine whether HSPG2 could be used as a marker of disease prognosis, the predictive capacity of HSPG2 levels was analyzed among AML cases and compared to BM blast count, an established prognostic marker for AML. As highlighted by the comparison of ROC curves (Fig. 3d), HSPG2 expression alone is enough to obtain high predictive accuracy for 2-year survival in whole-cohort AML (HSPG2 expression-AUC = 0.663, 95% CI: 0.572–0.754, *P* = 0.002), as compared with the predictive capacity of BM blasts (BM blasts-AUC = 0.613, 95% CI: 0.516–0.710, *P* = 0.034).

### Correlation of HSPG2 expression with clinical characteristics in AML

By the set cutoff value based on the ROC curve, the whole cohort of AML patients were divided into two groups, low HSPG2 expression (≤4.39) (HSPG2^low^) and high HSPG2 expression (>4.39) (HSPG2^high^). The comparisons of clinical manifestations and laboratory features between two groups were presented in Table 2. Interestingly, the high expression of HSPG2 was found to be associated with higher WBC (*P* < 0.001), lower hemoglobin (*P* = 0.002), lower platelets (*P* = 0.001), and higher BM blasts (*P* < 0.001). There were no significant differences in sex, age, French-American-British classification, karyotypes and treatment regimen between HSPG2^low^ and HSPG2^high^ patients. In addition, no significant correlations between HSPG2 expression and seven gene mutations were confirmed (*P* > 0.05).

**Table 2:** Comparisons of clinical manifestations and laboratory features in whole-cohort AML patients.

Patients parameters		HSPG2 expression		
	All patients (*n* = 151)	Low (*n* = 49)	High (*n* = 102)	*P*
Sex, male/female	78/73	26/23	52/50	0.863
Median age, year, (range)	48 (12–83)	42.5 (20–81)	50 (12–83)	0.287
Median WBC × 10^9^/L, (range)	5.19 (0.35–262.2)	3.97 (0.36–92.78)	8.47 (0.35–262.2)	<0.001
Median HGB, g/L, (range)	79 (11–183)	93.5 (41–183)	74 (11–148)	0.002
Median PLT, ×10^9^/L, (range)	56 (3–491)	105 (4–491)	42 (3–319)	0.001
BM blasts, % (range)	36 (0–96)	2 (0–91)	60 (1–96)	<0.001
FAB classification				0.933
M1	9/151 (6.0%)	2/49 (4.1%)	7/102 (6.9%)	
M2	44/151 (29.1%)	19/49 (38.8%)	25/102 (24.5%)	
M3	20/151 (13.2%)	4/49 (8.2%)	16/102 (15.7%)	
M4	20/151 (13.2%)	4/49 (8.2%)	16/102 (15.7%)	
M5	29/151 (19.2%)	11/49 (22.4%)	18/102 (17.6%)	
M6	4/151 (2.6%)	1/49 (2.0%)	3/102 (2.9%)	
M7	1/151 (0.7%)	0/49 (0.0%)	1/102 (1.0%)	
Others	7/151 (4.6%)	1/49 (2.0%)	6/102 (5.9%)	
sAML	17/151 (11.3%)	7/49 (14.3%)	10/102 (9.8%)	
Karyotypes				0.069
Normal	48/151 (31.8%)	26/49 (53.1%)	22/102 (21.6%)	
t (15;17)	6/151 (4.0%)	0/49 (0.0%)	6/102 (5.9%)	
t (8;21)	11/151 (7.3%)	5/49 (10.2%)	6/102 (5.9%)	
Other abnormal karyotypes	23/151 (15.2%)	8/49 (16.3%)	15/102 (14.7%)	
Complex karyotype (>3 chromosomal abnormalities)	5/151 (3.3%)	1/49 (2.0%)	4/102 (3.9%)	
No data	58/151 (38.4%)	9/49 (18.4%)	49/102 (48.0%)	
Gene mutation				
WT1	77/151 (51.0%)	30/49 (61.2%)	47/102 (46.1%)	0.086
NPM1	12/151 (7.9%)	4/49 (8.2%)	8/102 (7.8%)	1.000
DNMT3A	10/151 (6.6%)	2/49 (4.1%)	8/102 (7.8%)	0.501
C-KIT	11/151 (7.3%)	5/49 (10.2%)	6/102 (5.9%)	0.506
FLT3-ITD	7/151 (4.6%)	1/49 (2.0%)	6/102 (5.9%)	0.429
IDH1/2	17/151 (11.3%)	3/49 (6.1%)	14/102 (13.7%)	0.270
AML1-ETO	24/151 (15.9%)	12/49 (24.5%)	12/102 (11.8%)	0.058
Treatment regimen				
Chemotherapy-only	110/151 (72.8%)	31/49 (63.3%)	79/102 (77.5%)	0.066
HSCT	41/151 (27.2%)	18/49 (36.7%)	23/102 (22.5%)	
Response				
CR	70/151 (46.4%)	30/49 (61.2%)	40/102 (39.2%)	0.011

*WBC* white blood cells, *HGB* hemoglobin, *PLT* platelet, *FAB* French–American–British classification, *sAML* secondary acute myeloid leukemia, *HSCT* hematopoietic stem cell transplantation, *CR* complete remission.

### HSPG2 expression in the monitoring of AML

To investigate HSPG2 expression in the monitoring of AML, we detected HSPG2 expression in patients at different clinical stages (151 patients at diagnosis, 41 patients at the time of CR and 14 patients at the time of relapse). Notably, the expression level of HSPG2 was remarkably decreased in CR phase and then increased again in relapse phase (Fig. 2b). Moreover, CR rate was also significantly higher in HSPG2^low^ patients than in HSPG2^high^ patients (*P* = 0.011) (Table 2).

### Correlation between HSPG2 expression and clinical outcome

**Fig. 4:**
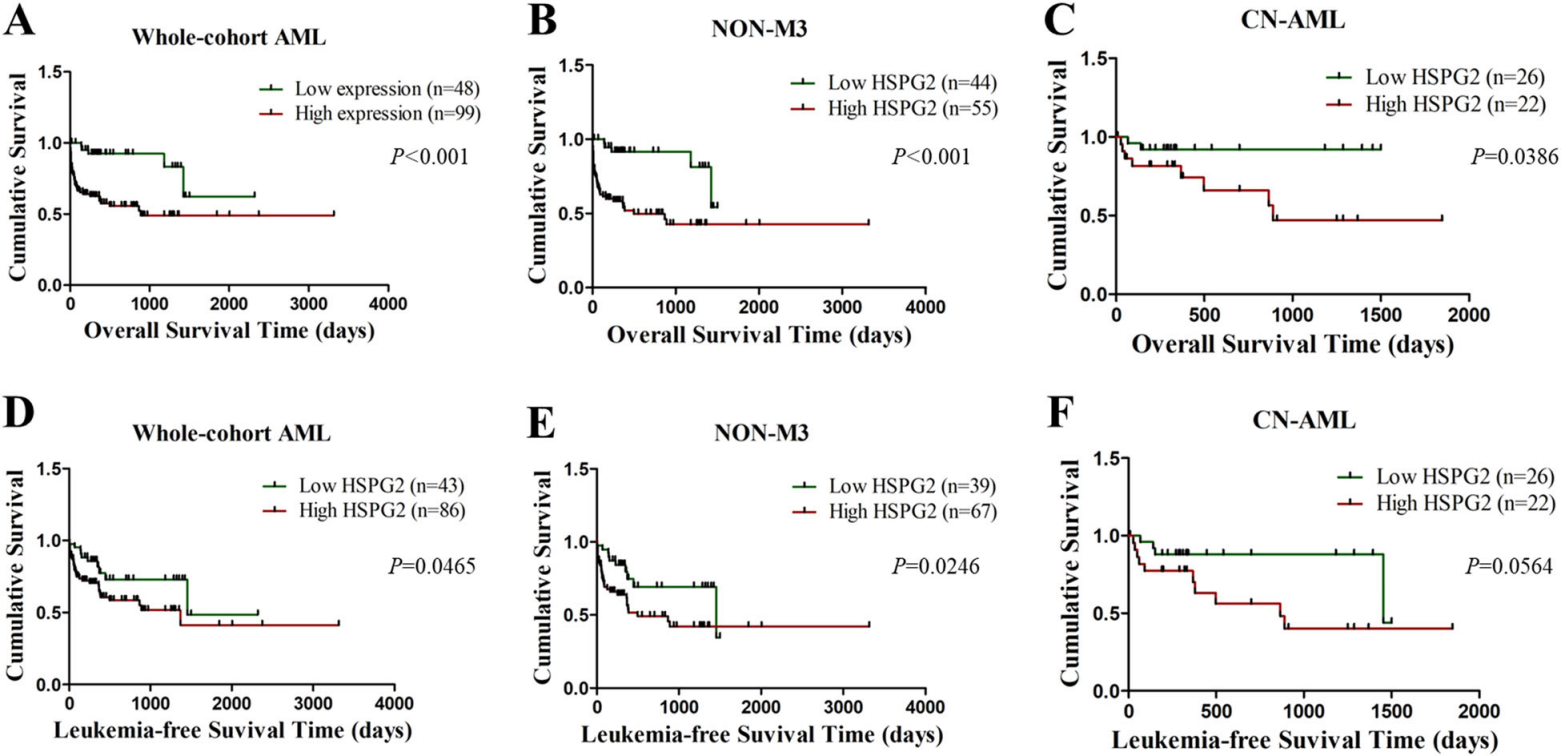
The impact of HSPG2 expression on survival in AML patients. **a** overall survival (OS) among whole-cohort AML. **b** OS among non-M3 AML. **c** OS among CN-AML. **d** leukemia-free survival (LFS) among whole-cohort AML. **e** LFS among non-M3 AML. **f** LFS among CN-AML. Non-M3 AML non M3 acute myeloid leukemia, CN-AML cytogenetically normal acute myeloid leukemia.

In this study, the median follow-up period was 9 months. Kaplan–Meier survival analysis manifested that OS and LFS of patients with high HSPG2 expression were significantly shorter than those of patients with low HSPG2 expression in the whole cohort of AML patients (*P* < 0.001, Fig. 4a; *P* = 0.047, Fig. 4d). Higher HSPG2 expression implied poor prognosis, which was also observed in the Non-M3 cohort (OS: *P* < 0.001, Fig. 4b; LFS: *P* = 0.025, Fig. 4e) and CN-AML patients (OS: *P* = 0.039, Fig. 4c; LFS: *P* = 0.056, Fig. 4f).

**Fig. 5:**
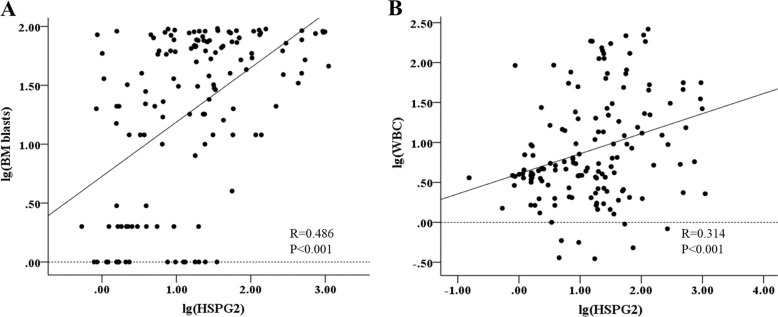
Spearman’s correlation analysis. **a** Between HSPG2 expression and BM blasts in AML patients. **b** Between HSPG2 expression and WBC count in AML patients. BM blasts bone marrow blasts, WBC white blood cell.

Univariate analyses and multivariate analyses on OS and LFS in the whole-cohort AML patients were performed, including the expression level of HSPG2, age (≥60 vs. <60 years old), WBC (≥30 × 10^9^/L vs. <30 × 10^9^/L), BM blasts(≥70% vs. <70%), treatment regimen and gene mutations (mutant vs. wild-type). As shown in Table 3, HSPG2 expression was significantly and independently associated with a worse OS both in univariate (*P* < 0.001) and multivariate analysis (*P* = 0.004). Moreover, HSPG2 expression was associated with a poorer LFS in univariate analysis (*P* = 0.047). Intriguingly, as shown in the results of Spearman’s rank correlation analysis (Fig. 5a, b), there was a positive correlation between HSPG2 expression and BM blasts (*r* = 0.486, *P* < 0.001), as well as WBC count (*r* = 0.314, *P* < 0.001).

**Table 3:** Univariate and multivariate analyses of prognostic factors for overall survival in whole-cohort AML patients.

Variables			OS				LFS	
	Univariate analysis		Multivariate analysis		Univariate analysis		Multivariate analysis	
	*P* value	HR (95% CI)	*P* value	HR (95% CI)	*P* value	HR (95% CI)	*P* value	HR (95% CI)
HSPG2 expression (high vs. low)	<0.001	4.683 (1.848–11.869)	0.004	3.963 (1.464–7.042)	0.047	2.025 (1.010–3.482)	0.302	–
Age (≥60 vs. <60)	0.005	2.338 (1.296–4.217)	0.798	–	0.214	1.465 (0.802–2.675)	–	–
Sex (male vs. female)	0.376	1.302 (0.726–2.332)	–	–	0.638	1.155 (0.634–2.105)	–	–
WBC (≥30 vs. <30)	0.001	2.831 (1.563–5.127)	0.113	–	0.004	2.550 (1.348–4.824)	0.026	1.969 (1.085–3.571)
BM Blasts (≥70% vs. <70%)	0.081	1.739 (0.935–3.236)	–	–	0.771	1.110 (0.552–2.232)	–	–
Treatment regimen	0.001	6.016 (2.146–16.866)	0.002	6.513 (1.969–21.542)	<0.001	5.672 (2.254–14.275)	0.001	5.793 (2.064–16.259)
WT1 mutation	0.358	0.761 (0.425–1.362)	–	–	0.779	1.090 (0.595–1.997)	–	–
NPM1 mutation	0.273	1.682 (0.663–4.264)	–	–	0.488	1.441 (0.514–4.040)	–	–
DNMT3A mutation	<0.001	4.799 (2.195–10.491)	0.004	3.922 (1.529–10.000)	<0.001	6.540 (2.942–14.538)	0.028	6.045 (1.100–5.263)
C-KIT mutation	0.445	0.574 (0.139–2.380)	–	–	0.542	0.641 (0.154–2.672)	–	–
FLT3-ITD mutation	0.089	2.443 (0.873–6.838)	–	–	0.015	3.194 (1.250–8.163)	0.986	–
IDH1/2 mutation	0.100	2.689 (0.827–8.804)	–	–	0.002	5.230 (1.836–14.897)	0.487	–
AML1-ETO mutation	0.026	0.200 (0.049–0.827)	–	–	0.021	0.096 (0.013–0.699)	0.09	–

*OS* overall survival, *LFS* leukemia-free survival, *HR* hazard ratio, *CI* confidence interval, *WBC* white blood cells, *BM* *Blasts* bone marrow blasts

Treatment regimen (with chemotherapy-only vs. with transplantation), gene mutations (mutant vs. wild-type).

## Discussion

Great progress has been obtained in our understanding of the pathogenesis of AML via the characterization of dysregulated genes, such as transcription factor SALL4^18^, ABC subfamily B-member 1 (ABCB1)^19^, and Homeodomain-only protein homeobox (HOPX)^20^. However, owing to the absence of an observation of the overall process before and after disease progression of AML, those known differential expression genes are still unable to illuminate the mechanism underlying disease progression and prognosis. The factors involved in disease progression and pathogenesis in AML remain to be elucidated. The five genes with a high upregulation and the five downregulation genes from our results of RNA-seq, according to their relevance to the pathogenesis and prognosis of cancers were selected for further investigation. ABCA13 has identified as a marker of poor survival in metastatic ovarian serous carcinoma^21^ and was found to be highly overexpressed in our results. ADD1 has been associated with primary hypertension^22^ and was also highly overexpressed. CCAR1 is a transcriptional coactivator for nuclear receptors and exerts its functions as a key intracellular signal transducer of apoptosis signaling pathways^23^, which was overexpressed in AML patients in this study. GLT1D1 was shown to be highly upregulated and promoted immunosuppression and tumor growth in B-cell non-Hodgkin’s lymphoma^24^. YY1 could be regulated by CXCR4 to mediate transcriptional activation of MYC and BCLXL in AML cells^25^. ATXN7 was found to decrease in AML patients in our cohort, and low expression of ATXN7 was significantly associated with poor recurrence-free survival (RFS) and OS in Hepatitis B Virus-Related Hepatocellular Carcinoma^26^. SET protein regulated intracellular redox state and sustained autophagy in Head and Neck Squamous Cell Carcinoma (HNSCC) cells, which may play an important role in resistance to the death of HNSCC cells^27^. The expression of CDH4 was decreased in nasopharyngeal carcinoma cell lines, xenografts and primary tumor biopsies and may be a novel putative tumor suppressor gene^28^. UXT is a novel regulator of the polycomb repressive complex 2 (PRC2) and acts as a renal cancer oncogene that affects the progression and survival of clear cell renal cell carcinoma patients^29^. Moreover, the dysregulation expression of HSPG2 was shown to affect the growth, invasion, metastasis, and angiogenesis of several tumors^30–32^.

In this study, we found that HSPG2 expression was elevated significantly in newly diagnosed AML patients compared with healthy controls, which was consistent with the results of RNA-seq. HSPG2 exists in various tissues and exerts effects on many biological processes^33^. The abnormal expression of HSPG2 has been reported in some carcinomas. In accordance with our findings, previous studies have found mRNA level of HSPG2 was markedly increased in solid tumor tissue compared with that in normal tissue, such as metastatic melanomas^34^, oral squamous carcinomas^35^ and hepatocellular carcinomas^36^. Particularly, our data showed that HSPG2 expression decreased after complete remission and returned during relapse phase. Consequently, it is considered that HSPG2 can play a vital role in disease progression and be a new molecular maker in AML. It is noteworthy that HSPG2 is significantly highly expressed in CD34^+^ cells of AML patients, suggesting that perlecan is indeed secreted by the hematopoietic stem/progenitor cells in AML, even though that perlecan is known to be produced from mesenchymal stem cells^37^. This observation, combined with our data that the expression of HSPG2 was higher in human myeloid leukemia cell lines than in bone marrow stromal cell line, explained why HSPG2 expression in AML patients was higher than that in healthy controls as mentioned above. Collectively, this study showed HSPG2 was significantly increased in AML patients, which revealed that inhibition of HSPG2 is a potential therapeutic strategy in AML.

In this study, the AUC value is preferable for HSPG2 expression to distinguish AML patients from healthy controls, including CN-AML and non-M3 AML, revealing it might be a promising diagnostic biomarker. The correlation between HSPG2 expression and several clinical and laboratory characteristics was then evaluated. One the one hand, high expression of HSPG2 was associated with lower hemoglobin and platelet count. On the other hand, high expression of HSPG2 was associated with higher WBC and BM blast count, which exhibited a positive correlation. A previous study demonstrated that high WBC count is a prognostic factor in patients with acute myeloid leukemia with the genotypic combination ‘NPMc (+) with FLT3-ITD’^38^. Guo RJ and coworkers^39^ also reported that high WBC count was an independent prognostic factor for a shorter OS and EFS. It is reported that excess blasts are the strongest predictors for poor outcome of AML and associated with the disease progression^40,41^. This discovery, combined with our findings that the ability of HSPG2 for predicting 2-year survival is similar to that of BM blasts in AML based on the AUC value and that there was a positive correlation between HSPG2 expression and BM blasts, suggested that HSPG2 expression might be a prognostic factor for AML. It is well known that cytogenetic abnormalities and somatic mutations have been found at diagnosis in 20–70% of AML patients and are critical in evaluating the outcome of AML patients^42^. Herein, we also explored whether the expression of HSPG2 was correlated with karyotypes and gene mutations. Unfortunately, no significant correlation was observed, which may be due to limited sample size.

In addition, we observed that CR rate was significantly lower in HSPG2^high^ patients after receiving induction chemotherapy, which indicated that HSPG2 expression might be related to the response to the treatment of AML. Furthermore, survival analysis revealed that patients with high HSPG2 expression had a poor prognosis in whole cohort AML, non-M3 AML and CN-AML. We also found that HSPG2 was an independent prognostic factor for OS based on univariate and multivariate analyses and for LFS based on univariate analysis. As reported in oligoastrocytoma and oligodendroglioma, high expression of HSPG2 could independently predict poor OS and RFS^13^. Hence, HSPG2 expression could be used to predict inferior survival and assess treatment outcome in AML. It is worth mentioning the treatment regimen is a crucial factor for OS and LFS in AML and we found that patients treated with hematopoietic stem cell transplantation (HSCT) had better OS and LFS compared with chemotherapy alone. Based on these results, we can draw the conclusion that the expression level of HSPG2 could be used as an important indicator of prognosis in AML. Certainly, more in-depth studies on large-scale samples of AML patients are needed to verify these findings.

This study implied that the expression level of HSPG2 was upregulated in AML patients and correlated with adverse prognosis and disease progression in AML patients. To sum up, HSPG2 potentially might act as a pro-oncogene in the pathogenesis of AML, and further functional researches should be conducted to confirm its role in vivo and in vitro.

## Conclusion

In summary, this is the first study to report the relationship of HSPG2 expression with the clinical outcomes in AML patients. This study confirmed that overexpression of HSPG2 was an adverse prognostic indicator for AML, although the mechanism underlying the role of HSPG2 in AML remains to be illustrated.

## Supplementary information

Table S1

Fig. S1
