# Supplementary material for: HSPG2 overexpression independently predicts poor survival in patients with acute myeloid leukemia
Source: Cell Death Dis. 2020 Jun 30;11(6):492. doi: 10.1038/s41419-020-2694-7 (PMC7327006; doi:10.1038/s41419-020-2694-7)
Supplement: Supplementary file 1 — Table S1 [file 41419_2020_2694_MOESM1_ESM.doc]

**Supplementary Table S1.**

| **No.** | **Genes** | **Log_2_ Fold Change** | ***p*-value** | ***Qvalue*** | ***updown*** |
| --- | --- | --- | --- | --- | --- |
| **Up-regulated** |  |  |  |  |  |
| 1 | CDH4 | Inf | 1.32E-09 | 7.60E-06 | UP |
| 2 | ATXN7 | 9.846778 | 3.54E-07 | 0.001163 | UP |
| 3 | OBSL1 | Inf | 7.77E-07 | 0.001948 | UP |
| 4 | PHF21A | Inf | 9.97E-07 | 0.002376 | UP |
| 5 | MEF2C | Inf | 1.51E-06 | 0.00293 | UP |
| 6 | DROSHA | Inf | 1.88E-06 | 0.003505 | UP |
| 7 | ZBTB21 | Inf | 3.13E-06 | 0.00523 | UP |
| 8 | ZNF185 | Inf | 4.44E-06 | 0.006507 | UP |
| 9 | AC009053.1 | Inf | 6.25E-06 | 0.007546 | UP |
| 10 | RBM48 | Inf | 7.02E-06 | 0.008064 | UP |
| 11 | WDR61 | Inf | 7.54E-06 | 0.008354 | UP |
| 12 | PDPR | Inf | 8.15E-06 | 0.008829 | UP |
| 13 | NBPF12 | Inf | 9.34E-06 | 0.009781 | UP |
| 14 | PNKD | Inf | 1.23E-05 | 0.01128 | UP |
| 15 | FDFT1 | Inf | 1.24E-05 | 0.01128 | UP |
| 16 | RNMT | Inf | 1.37E-05 | 0.011853 | UP |
| 17 | GUSBP1 | Inf | 1.68E-05 | 0.014175 | UP |
| 18 | SET | 7.902441 | 1.71E-05 | 0.014227 | UP |
| 19 | UXT | 7.092185 | 1.91E-05 | 0.014918 | UP |
| 20 | HIST1H4E | 8.605986 | 2.36E-05 | 0.017729 | UP |
| 21 | PUM2 | 8.686447 | 2.73E-05 | 0.019859 | UP |
| 22 | CLSTN3 | Inf | 2.84E-05 | 0.020535 | UP |
| 23 | KMT2E | Inf | 3.39E-05 | 0.023433 | UP |
| 24 | PHF23 | Inf | 3.61E-05 | 0.024432 | UP |
| 25 | HLA-A | 10.08128 | 3.77E-05 | 0.025323 | UP |
| 26 | TRIM26 | Inf | 4.01E-05 | 0.026347 | UP |
| 27 | ANKRD36 | Inf | 4.51E-05 | 0.029289 | UP |
| 28 | FABP5 | 9.514594 | 5.13E-05 | 0.032201 | UP |
| 29 | SRSF5 | 8.796784 | 5.35E-05 | 0.033109 | UP |
| 30 | XRCC6 | Inf | 5.59E-05 | 0.03374 | UP |
| 31 | KAT6B | Inf | 6.69E-05 | 0.039544 | UP |
| 32 | VPS41 | Inf | 7.40E-05 | 0.04123 | UP |
| 33 | STAR | Inf | 7.27E-05 | 0.04123 | UP |
| 34 | 9-Sep | Inf | 7.87E-05 | 0.042981 | UP |
| 35 | TBC1D1 | Inf | 8.20E-05 | 0.043405 | UP |
| 36 | RASSF1 | Inf | 8.46E-05 | 0.043854 | UP |
| 37 | ANGPT1 | Inf | 8.64E-05 | 0.044177 | UP |
| 38 | ABR | 8.345163 | 9.64E-05 | 0.047631 | UP |
| 39 | SCAP | Inf | 9.73E-05 | 0.047819 | UP |
| 40 | RAB37 | Inf | 9.85E-05 | 0.047976 | UP |
| 41 | CELF2 | Inf | 9.86E-05 | 0.047976 | UP |
| 42 | PSMC5 | 9.276996 | 0.000103 | 0.04971 | UP |
| 43 | HSPG2 | 3.36530722 | 1.94E-05 | 0.008003168 | UP |
| 44 | RP11-366M4.3 | 2.05705 | 0.012 | >0.05 | UP |
| 45 | CNTFR | 3.90651 | 0.0476 | >0.05 | UP |
| 46 | CRABP2 | 1.99188 | 0.03615 | >0.05 | UP |
| 47 | MYEOV | 1.90743 | 0.00525 | >0.05 | UP |
| 48 | DCX | 2.35224 | 0.0064 | >0.05 | UP |
| 49 | RAB32 | 2.55056 | 0.03425 | >0.05 | UP |
| 50 | SORL1 | 4.52615 | 0.0117 | >0.05 | UP |
| 51 | AKAP12 | 1.73198 | 0.0001 | >0.05 | UP |
| 52 | CFH | 1.95871 | 0.00335 | >0.05 | UP |
| 53 | B4GALT1 | 1.80287 | 0.0001 | >0.05 | UP |
| 54 | TMEM215 | 4.30075 | 0.03435 | >0.05 | UP |
| 55 | NFE2L3 | 1.99397 | 0.0077 | >0.05 | UP |
| 56 | MAGEC1 | 2.14764 | 0.0044 | >0.05 | UP |
| 57 | SIX3 | 2.98316 | 0.01755 | >0.05 | UP |
| 58 | MAP3K8 | 2.8109 | 0.0384 | >0.05 | UP |
| 59 | TNFRSF4 | inf | 0.00095 | >0.05 | UP |
| 60 | LRRC20 | 12.6786 | 0.04055 | >0.05 | UP |
| 61 | HCG22 | 1.88899 | 0.03615 | >0.05 | UP |
| 62 | CFL1 | inf | 0.04505 | >0.05 | UP |
| 63 | MYLK | 1.77715 | 0.00025 | >0.05 | UP |
| 64 | PITPNB | 1.76266 | 0.0455 | >0.05 | UP |
| 65 | WWC3 | 2.01996 | 0.0005 | >0.05 | UP |
| 66 | DDIT4 | 1.69051 | 0.005 | >0.05 | UP |
| 67 | FBXO44 | 13.0416 | 0.0492 | >0.05 | UP |
| 68 | MMP2 | 13.6455 | 0.0287 | >0.05 | UP |
| 69 | TBX3 | 1.94141 | 0.0458 | >0.05 | UP |
| 70 | APP | 1.47412 | 0.04865 | >0.05 | UP |
| 71 | RYR2 | 2.10743 | 0.00085 | >0.05 | UP |
| 72 | MYADM | 13.2428 | 0.03975 | >0.05 | UP |
| 73 | TMEM178B | 1.57051 | 0.0002 | >0.05 | UP |
| 74 | FBLN1 | 1.45855 | 0.00195 | >0.05 | UP |
| 75 | RP11-497H16.7 | inf | 0.02615 | >0.05 | UP |
| 76 | IFNGR2 | 1.5083 | 0.02025 | >0.05 | UP |
| 77 | JAK3 | 2.22437 | 0.0132 | >0.05 | UP |
| 78 | WIPF3 | 2.23009 | 0.0214 | >0.05 | UP |
| 79 | CA8 | inf | 0.00135 | >0.05 | UP |
| 80 | TIFA | 1.76786 | 0.0241 | >0.05 | UP |
| 81 | KCNB2 | inf | 0.00005 | >0.05 | UP |
| 82 | HP1BP3 | 1.54847 | 0.03875 | >0.05 | UP |
| 83 | KIAA1671 | 1.83896 | 0.04165 | >0.05 | UP |
| 84 | JUN | 1.60125 | 0.00365 | >0.05 | UP |
| 85 | FAM73A | 1.56624 | 0.0015 | >0.05 | UP |
| 86 | TAP1 | 1.83736 | 0.0268 | >0.05 | UP |
| 87 | LRRC37A3 | 13.2133 | 0.04305 | >0.05 | UP |
| 88 | DNAJA1 | 1.51362 | 0.00005 | >0.05 | UP |
| 89 | RAX | 1.78893 | 0.01275 | >0.05 | UP |
| 90 | VARS2 | 12.4913 | 0.04875 | >0.05 | UP |
| 91 | SPATA13 | 1.58447 | 0.0032 | >0.05 | UP |
| 92 | ARNT2 | 2.88821 | 0.00445 | >0.05 | UP |
| 93 | TMSB4X | 1.41226 | 0.0013 | >0.05 | UP |
| 94 | SIX4 | 1.88489 | 0.0056 | >0.05 | UP |
| 95 | ATP11A | 1.88978 | 0.0468 | >0.05 | UP |
| 96 | MYPN | 2.17979 | 0.0263 | >0.05 | UP |
| 97 | TNFRSF21 | 2.0263 | 0.03415 | >0.05 | UP |
| 98 | GFRA2 | 1.75401 | 0.04285 | >0.05 | UP |
| 99 | PPP1R14C | 2.53214 | 0.03175 | >0.05 | UP |
| 100 | CPNE8 | 3.05924 | 0.0165 | >0.05 | UP |
| 101 | MYO16 | 3.82674 | 0.02305 | >0.05 | UP |
| 102 | NFKBIE | 1.55581 | 0.0405 | >0.05 | UP |
| 103 | RP11-215G15.5 | 1.94825 | 0.0399 | >0.05 | UP |
| 104 | AC022816.2 | inf | 0.0008 | >0.05 | UP |
| 105 | HS6ST2 | 1.64436 | 0.0186 | >0.05 | UP |
| 106 | HIPK2 | 1.46316 | 0.0004 | >0.05 | UP |
| 107 | OAS3 | 1.38112 | 0.00075 | >0.05 | UP |
| 108 | ZNF849P | 13.3616 | 0.03435 | >0.05 | UP |
| 109 | HUNK | 3.06196 | 0.0166 | >0.05 | UP |
| 110 | HMX2 | inf | 0.00015 | >0.05 | UP |
| 111 | BHLHE40 | 3.63925 | 0.0389 | >0.05 | UP |
| 112 | FOXO1 | 2.41048 | 0.0158 | >0.05 | UP |
| 113 | CDK11B | 1.43283 | 0.0351 | >0.05 | UP |
| 114 | LIMCH1 | 14.5934 | 0.02705 | >0.05 | UP |
| 115 | MYCBP2 | 1.39805 | 0.01395 | >0.05 | UP |
| 116 | CACHD1 | 1.25834 | 0.02355 | >0.05 | UP |
| 117 | TRAF3 | 1.24306 | 0.00595 | >0.05 | UP |
| 118 | UST | 1.66285 | 0.0495 | >0.05 | UP |
| 119 | DCHS2 | 3.96101 | 0.00375 | >0.05 | UP |
| 120 | HMGB1P5 | 1.19398 | 0.0242 | >0.05 | UP |
| 121 | CNTNAP2 | 1.19903 | 0.0015 | >0.05 | UP |
| 122 | SMU1 | 1.19915 | 0.04925 | >0.05 | UP |
| 123 | ZFAND2B | 14.1545 | 0.047 | >0.05 | UP |
| 124 | ETS2 | 1.2443 | 0.00495 | >0.05 | UP |
| 125 | FRK | 2.65514 | 0.004 | >0.05 | UP |
| 126 | PALB2 | 13.6014 | 0.0431 | >0.05 | UP |
| 127 | MSRB3 | 14.3572 | 0.04095 | >0.05 | UP |
| 128 | IFNAR1 | 1.32152 | 0.01075 | >0.05 | UP |
| 129 | BICC1 | 2.54826 | 0.0277 | >0.05 | UP |
| 130 | MAB21L3 | 1.45979 | 0.0439 | >0.05 | UP |
| 131 | ADAMTS1 | 1.43536 | 0.01185 | >0.05 | UP |
| 132 | DLX3 | 1.4537 | 0.0363 | >0.05 | UP |
| 133 | SYT1 | 1.2991 | 0.0022 | >0.05 | UP |
| 134 | EGFR | inf | 0.00005 | >0.05 | UP |
| 135 | FAM214B | 14.8358 | 0.0231 | >0.05 | UP |
| 136 | TLR7 | inf | 0.00005 | >0.05 | UP |
| 137 | DSP | 1.96756 | 0.00775 | >0.05 | UP |
| 138 | SOX2 | 1.2451 | 0.00425 | >0.05 | UP |
| 139 | RGL1 | 1.28925 | 0.00495 | >0.05 | UP |
| 140 | HLA-A | 1.18232 | 0.0244 | >0.05 | UP |
| 141 | YPEL5 | 1.14621 | 0.01415 | >0.05 | UP |
| 142 | MAGED4 | 11.0092 | 0.03885 | >0.05 | UP |
| 143 | PARP14 | 1.32917 | 0.0149 | >0.05 | UP |
| 144 | USP40 | 1.27902 | 0.0147 | >0.05 | UP |
| 145 | ENPP4 | 1.43416 | 0.02845 | >0.05 | UP |
| 146 | ALK | 1.54101 | 0.0233 | >0.05 | UP |
| 147 | ZNF221 | 15.6167 | 0.0387 | >0.05 | UP |
| 148 | SMAD9 | 1.26464 | 0.045 | >0.05 | UP |
| 149 | SDC4 | 1.22582 | 0.0422 | >0.05 | UP |
| 150 | CYYR1 | 1.11093 | 0.00345 | >0.05 | UP |
| 151 | C2CD2 | 1.37119 | 0.02155 | >0.05 | UP |
| 152 | VCP | 1.10828 | 0.0044 | >0.05 | UP |
| 153 | LRCH1 | 1.23914 | 0.0156 | >0.05 | UP |
| 154 | KIAA1549 | 1.82557 | 0.0162 | >0.05 | UP |
| 155 | MGAT3 | 1.36512 | 0.02365 | >0.05 | UP |
| 156 | DNAJC12 | 1.13165 | 0.0173 | >0.05 | UP |
| 157 | ABCA5 | 15.3273 | 0.03835 | >0.05 | UP |
| 158 | CSAG2 | inf | 0.02155 | >0.05 | UP |
| 159 | AC079753.4 | inf | 0.0274 | >0.05 | UP |
| 160 | RP1-104O17.3 | inf | 0.0224 | >0.05 | UP |
| 161 | CRYGN | inf | 0.03965 | >0.05 | UP |
| 162 | RP11-122C5.1 | inf | 0.022 | >0.05 | UP |
| 163 | LINC00871 | inf | 0.0186 | >0.05 | UP |
| 164 | PLA2G4C | inf | 0.04625 | >0.05 | UP |
| 165 | RP11-540O11.1 | inf | 0.03575 | >0.05 | UP |
| 166 | RP11-757O6.4 | inf | 0.03575 | >0.05 | UP |
| 167 | RP11-62H20.1 | inf | 0.03655 | >0.05 | UP |
| 168 | CTD-2619J13.16 | inf | 0.03575 | >0.05 | UP |
| 169 | RP11-425D10.10 | inf | 0.01495 | >0.05 | UP |
| 170 | RP11-89C3.3 | inf | 0.03575 | >0.05 | UP |
| 171 | SPINK2 | inf | 0.0306 | >0.05 | UP |
| 172 | OR7E101P | inf | 0.04305 | >0.05 | UP |
| 173 | RP11-599B13.3 | inf | 0.03575 | >0.05 | UP |
| 174 | AC008746.12 | inf | 0.03575 | >0.05 | UP |
| 175 | FCGBP | 1.74474 | 0.0358 | >0.05 | UP |
| 176 | PCDHB14 | 13.0804 | 0.0361 | >0.05 | UP |
| 177 | CHI3L1 | inf | 0.005 | >0.05 | UP |
| 178 | RLN2 | inf | 0.0218 | >0.05 | UP |
| 179 | RP11-269F21.3 | inf | 0.0279 | >0.05 | UP |
| 180 | HSD17B14 | inf | 0.008 | >0.05 | UP |
| 181 | RP1-302G2.5 | inf | 0.02115 | >0.05 | UP |
| 182 | RP11-474I16.8 | inf | 0.0274 | >0.05 | UP |
| 183 | AC137934.1 | inf | 0.01715 | >0.05 | UP |
| 184 | RP11-345K20.2 | inf | 0.03575 | >0.05 | UP |
| 185 | LINC00882 | inf | 0.01875 | >0.05 | UP |
| 186 | RP11-70P17.1 | inf | 0.0274 | >0.05 | UP |
| 187 | RP11-707G14.7 | inf | 0.03575 | >0.05 | UP |
| 188 | C6orf58 | inf | 0.0012 | >0.05 | UP |
| 189 | PKM | inf | 0.0446 | >0.05 | UP |
| 190 | IL12RB2 | 14.2544 | 0.03085 | >0.05 | UP |
| 191 | SULT1A3 | 10.8325 | 0.04695 | >0.05 | UP |
| 192 | RP11-421N8.1 | inf | 0.0274 | >0.05 | UP |
| 193 | MAPK11 | 14.9081 | 0.0331 | >0.05 | UP |
| 194 | DAP3P2 | inf | 0.0274 | >0.05 | UP |
| 195 | CCDC160 | inf | 0.0063 | >0.05 | UP |
| 196 | TUBA1B | inf | 0.02905 | >0.05 | UP |
| 197 | KLHL2P1 | inf | 0.0282 | >0.05 | UP |
| 198 | RP11-575H3.1 | inf | 0.0274 | >0.05 | UP |
| 199 | CTC-360P9.3 | inf | 0.02785 | >0.05 | UP |
| 200 | FUT9 | 1.39428 | 0.04795 | >0.05 | UP |
| 201 | MPDU1 | inf | 0.04885 | >0.05 | UP |
| 202 | RSL24D1P6 | inf | 0.03575 | >0.05 | UP |
| 203 | IGHV3-49 | inf | 0.022 | >0.05 | UP |
| 204 | COL12A1 | 3.52109 | 0.02105 | >0.05 | UP |
| 205 | FGFR1OP | 1.1284 | 0.0337 | >0.05 | UP |
| 206 | EMB | inf | 0.00005 | >0.05 | UP |
| 207 | ADAL | 13.2459 | 0.0395 | >0.05 | UP |
| 208 | CLCN2 | inf | 0.0464 | >0.05 | UP |
| 209 | HIVEP1 | 1.0804 | 0.03525 | >0.05 | UP |
| 210 | RP11-524D16__A.3 | inf | 0.0139 | >0.05 | UP |
| 211 | SLC25A13 | 11.76 | 0.04205 | >0.05 | UP |
| 212 | PLCB4 | 15.2309 | 0.0305 | >0.05 | UP |
| 213 | JAG1 | 1.88411 | 0.0421 | >0.05 | UP |
| 214 | STC1 | 1.06379 | 0.04745 | >0.05 | UP |
| 215 | ZIC5 | 1.05444 | 0.03225 | >0.05 | UP |
| 216 | OR2I1P | inf | 0.0022 | >0.05 | UP |
| 217 | FAM131A | inf | 0.04675 | >0.05 | UP |
| 218 | IRS2 | 1.01041 | 0.00945 | >0.05 | UP |
| 219 | AHDC1 | 1.11337 | 0.0339 | >0.05 | UP |
| 220 | RP11-51F16.9 | inf | 0.022 | >0.05 | UP |
| 221 | FBN2 | 1.03043 | 0.0146 | >0.05 | UP |
| 222 | AC016907.3 | inf | 0.02205 | >0.05 | UP |
| 223 | NEDD9 | 2.55427 | 0.035 | >0.05 | UP |
| 224 | NOL6 | 1.04924 | 0.0069 | >0.05 | UP |
| 225 | NCBP2 | 1.02918 | 0.025 | >0.05 | UP |
| 226 | SCNN1G | 1.06806 | 0.0135 | >0.05 | UP |
| 227 | RP11-316P17.2 | inf | 0.0014 | >0.05 | UP |
| 228 | CBFA2T2 | 13.4467 | 0.04605 | >0.05 | UP |
| 229 | HSPA12A | 2.97913 | 0.04415 | >0.05 | UP |
| 230 | CHMP5 | 1.01098 | 0.01685 | >0.05 | UP |
| 231 | KDM8 | 12.2933 | 0.04195 | >0.05 | UP |
| 232 | TRIM69 | 1.06048 | 0.03085 | >0.05 | UP |
| 233 | TOLLIP | 1.11623 | 0.04475 | >0.05 | UP |
| 234 | DMXL1 | 13.9382 | 0.023 | >0.05 | UP |
| 235 | MARCKS | 1.13376 | 0.0177 | >0.05 | UP |
| 236 | ZNF462 | 1.95268 | 0.022 | >0.05 | UP |
| 237 | KCNE4 | 1.13057 | 0.0292 | >0.05 | UP |
| 238 | SLC25A30 | 1.09825 | 0.00965 | >0.05 | UP |
| 239 | ACO1 | 1.11142 | 0.02105 | >0.05 | UP |
| 240 | COTL1 | 1.08236 | 0.01895 | >0.05 | UP |
| 241 | PIM3 | 1.08116 | 0.01725 | >0.05 | UP |
| 242 | CAV1 | 1.04173 | 0.0098 | >0.05 | UP |
| 243 | HLA-DRA | inf | 0.0006 | >0.05 | UP |
| 244 | TMEM2 | 1.4771 | 0.0377 | >0.05 | UP |
| 245 | RNF38 | 1.21849 | 0.0408 | >0.05 | UP |
| 246 | WASH2P | 13.8817 | 0.04605 | >0.05 | UP |
| 247 | KB-1027C11.4 | inf | 0.01495 | >0.05 | UP |
| Down-regulated |  |  |  |  |  |
| 248 | CCAR1 | #NAME? | 2.88E-10 | 5.05E-06 | DOWN |
| 249 | GLT1D1 | #NAME? | 1.08E-09 | 1.03E-05 | DOWN |
| 250 | ADD1 | -9.586281487 | 1.20E-07 | 0.000474742 | DOWN |
| 251 | YY1 | #NAME? | 7.18E-07 | 0.001901227 | DOWN |
| 252 | ABCA13 | -6.991039081 | 6.49E-05 | 0.038658258 | DOWN |
| 253 | NCOA3 | -12.5922 | 8.02E-12 | 7.64E-07 | DOWN |
| 254 | KNL1 | -1.88987 | 7.21E-11 | 1.72E-06 | DOWN |
| 255 | ANKHD1 | -2.46246 | 7.10E-11 | 1.72E-06 | DOWN |
| 256 | TROVE2 | -2.58915 | 6.36E-11 | 1.72E-06 | DOWN |
| 257 | BICD2 | -1.33501 | 3.18E-10 | 5.05E-06 | DOWN |
| 258 | MGAT4A | -1.46038 | 7.19E-10 | 7.97E-06 | DOWN |
| 259 | ZHX1 | -2.36301 | 6.52E-10 | 7.97E-06 | DOWN |
| 260 | TRIQK | -13.0462 | 7.52E-10 | 7.97E-06 | DOWN |
| 261 | ATAD5 | -1.71854 | 2.56E-09 | 2.22E-05 | DOWN |
| 262 | MTMR14 | -11.9206 | 3.40E-09 | 2.69E-05 | DOWN |
| 263 | ATP5A1 | #NAME? | 3.67E-09 | 2.69E-05 | DOWN |
| 264 | STAG2 | -1.3939 | 1.18E-08 | 8.05E-05 | DOWN |
| 265 | PIP5K1B | -12.3508 | 1.34E-08 | 8.54E-05 | DOWN |
| 266 | TACC1 | -13.2581 | 2.12E-08 | 0.000126 | DOWN |
| 267 | VCAM1 | -2.78154 | 3.48E-08 | 0.000175 | DOWN |
| 268 | DEPDC1 | -1.62693 | 3.22E-08 | 0.000175 | DOWN |
| 269 | FANCD2 | -13.2421 | 3.40E-08 | 0.000175 | DOWN |
| 270 | PREPL | -1.81715 | 3.79E-08 | 0.000181 | DOWN |
| 271 | MARK2 | -3.70144 | 5.55E-08 | 0.000252 | DOWN |
| 272 | BANK1 | -1.42367 | 8.28E-08 | 0.000359 | DOWN |
| 273 | VDR | #NAME? | 1.04E-07 | 0.000432 | DOWN |
| 274 | SMG7 | -2.67324 | 1.40E-07 | 0.000533 | DOWN |
| 275 | SLC1A4 | -1.70375 | 1.52E-07 | 0.000557 | DOWN |
| 276 | CENPE | #NAME? | 2.55E-07 | 0.000901 | DOWN |
| 277 | ABRAXAS1 | -1.53892 | 3.48E-07 | 0.001163 | DOWN |
| 278 | CALM1 | -1.78492 | 5.50E-07 | 0.001692 | DOWN |
| 279 | ATRX | -1.53826 | 5.98E-07 | 0.001783 | DOWN |
| 280 | ISCU | -14.7093 | 6.45E-07 | 0.001863 | DOWN |
| 281 | TYK2 | -1.68503 | 6.82E-07 | 0.001876 | DOWN |
| 282 | MIER1 | -1.50271 | 6.89E-07 | 0.001876 | DOWN |
| 283 | POLE2 | -1.51297 | 7.40E-07 | 0.001907 | DOWN |
| 284 | PSMD1 | -12.7783 | 8.87E-07 | 0.002169 | DOWN |
| 285 | KIDINS220 | -1.49672 | 1.05E-06 | 0.002441 | DOWN |
| 286 | RPS18 | -14.8535 | 1.08E-06 | 0.002454 | DOWN |
| 287 | MRFAP1 | -1.60754 | 1.19E-06 | 0.002629 | DOWN |
| 288 | PPT1 | -4.00685 | 1.25E-06 | 0.002702 | DOWN |
| 289 | ATRN | -1.60808 | 1.30E-06 | 0.002748 | DOWN |
| 290 | SUMO1 | -1.84065 | 1.44E-06 | 0.002869 | DOWN |
| 291 | SMARCA4 | #NAME? | 1.41E-06 | 0.002869 | DOWN |
| 292 | PTMA | -1.66977 | 1.81E-06 | 0.003449 | DOWN |
| 293 | PTGR1 | -2.22283 | 2.26E-06 | 0.004142 | DOWN |
| 294 | SULF2 | -2.18102 | 2.33E-06 | 0.004189 | DOWN |
| 295 | SSR1 | -1.57924 | 2.47E-06 | 0.004361 | DOWN |
| 296 | SLC25A32 | -1.10365 | 2.79E-06 | 0.004832 | DOWN |
| 297 | RGPD1 | -1.08365 | 3.23E-06 | 0.005307 | DOWN |
| 298 | ARFGAP2 | -1.09231 | 3.50E-06 | 0.005656 | DOWN |
| 299 | ITGB3BP | -2.23763 | 3.69E-06 | 0.005861 | DOWN |
| 300 | CUL2 | -1.19959 | 3.82E-06 | 0.005971 | DOWN |
| 301 | ZNF747 | -1.11767 | 4.03E-06 | 0.00619 | DOWN |
| 302 | CEACAM3 | -1.16789 | 4.21E-06 | 0.006368 | DOWN |
| 303 | OXR1 | -2.62134 | 4.63E-06 | 0.006583 | DOWN |
| 304 | NCAPD3 | -13.3004 | 4.59E-06 | 0.006583 | DOWN |
| 305 | SLC4A7 | -1.38112 | 4.72E-06 | 0.006611 | DOWN |
| 306 | BLM | -1.34189 | 4.78E-06 | 0.006611 | DOWN |
| 307 | GYG1 | -15.4931 | 4.89E-06 | 0.006665 | DOWN |
| 308 | ANKRD36B | -1.28407 | 5.38E-06 | 0.006856 | DOWN |
| 309 | PGM2 | -1.61266 | 5.34E-06 | 0.006856 | DOWN |
| 310 | SREBF2 | -2.19131 | 5.38E-06 | 0.006856 | DOWN |
| 311 | WASHC2C | -1.2452 | 5.40E-06 | 0.006856 | DOWN |
| 312 | NCAPG2 | -1.19866 | 6.13E-06 | 0.007489 | DOWN |
| 313 | CCHCR1 | -1.18368 | 6.11E-06 | 0.007489 | DOWN |
| 314 | TNIP1 | -1.20434 | 6.43E-06 | 0.007659 | DOWN |
| 315 | STX16 | #NAME? | 7.06E-06 | 0.008064 | DOWN |
| 316 | ZGRF1 | -1.94185 | 6.91E-06 | 0.008064 | DOWN |
| 317 | LUC7L2 | -1.25689 | 7.11E-06 | 0.008064 | DOWN |
| 318 | KAT6B | -1.14978 | 7.79E-06 | 0.00854 | DOWN |
| 319 | RIPOR2 | -1.11191 | 8.82E-06 | 0.00945 | DOWN |
| 320 | CARD8 | -15.5208 | 9.51E-06 | 0.00985 | DOWN |
| 321 | CEP152 | -15.8089 | 9.94E-06 | 0.010159 | DOWN |
| 322 | ZNF558 | -1.18859 | 1.08E-05 | 0.010619 | DOWN |
| 323 | RRAGD | -1.21334 | 1.07E-05 | 0.010619 | DOWN |
| 324 | LTF | -12.9742 | 1.07E-05 | 0.010619 | DOWN |
| 325 | MR1 | -12.4566 | 1.17E-05 | 0.011255 | DOWN |
| 326 | VAPB | -12.8068 | 1.18E-05 | 0.011255 | DOWN |
| 327 | BRIP1 | -11.6507 | 1.18E-05 | 0.011255 | DOWN |
| 328 | CASP8 | -1.05951 | 1.22E-05 | 0.01128 | DOWN |
| 329 | RHOT1 | -1.29596 | 1.27E-05 | 0.01128 | DOWN |
| 330 | CEACAM1 | #NAME? | 1.24E-05 | 0.01128 | DOWN |
| 331 | CAMKK2 | -1.06307 | 1.26E-05 | 0.01128 | DOWN |
| 332 | BID | -1.12877 | 1.26E-05 | 0.01128 | DOWN |
| 333 | SYNE2 | -2.50311 | 1.28E-05 | 0.011325 | DOWN |
| 334 | USP25 | -14.6132 | 1.56E-05 | 0.013401 | DOWN |
| 335 | EIF2A | -14.8995 | 1.67E-05 | 0.014175 | DOWN |
| 336 | RRM2 | -1.28728 | 1.73E-05 | 0.014227 | DOWN |
| 337 | PRC1 | -1.22505 | 1.72E-05 | 0.014227 | DOWN |
| 338 | SLC9B2 | -11.9812 | 1.78E-05 | 0.014265 | DOWN |
| 339 | POGZ | -1.3329 | 1.75E-05 | 0.014265 | DOWN |
| 340 | RBBP4 | -1.39082 | 1.78E-05 | 0.014265 | DOWN |
| 341 | AK1 | -2.25836 | 1.82E-05 | 0.014486 | DOWN |
| 342 | UPF3B | -1.69489 | 1.89E-05 | 0.014881 | DOWN |
| 343 | TBC1D14 | -4.4252 | 1.95E-05 | 0.01509 | DOWN |
| 344 | CKAP5 | #NAME? | 2.06E-05 | 0.015853 | DOWN |
| 345 | RBL1 | -2.11149 | 2.36E-05 | 0.017729 | DOWN |
| 346 | IQGAP1 | -14.0659 | 2.46E-05 | 0.018351 | DOWN |
| 347 | MTF2 | -1.65146 | 2.59E-05 | 0.019054 | DOWN |
| 348 | LTN1 | -13.1769 | 2.60E-05 | 0.019054 | DOWN |
| 349 | CCDC25 | -1.75631 | 2.95E-05 | 0.021181 | DOWN |
| 350 | RFWD2 | -3.69039 | 3.03E-05 | 0.021418 | DOWN |
| 351 | EIF4E3 | -2.86805 | 3.02E-05 | 0.021418 | DOWN |
| 352 | ZNF3 | -1.83164 | 3.16E-05 | 0.022184 | DOWN |
| 353 | LAP3 | -2.27815 | 3.36E-05 | 0.023349 | DOWN |
| 354 | FANCI | -2.23345 | 3.57E-05 | 0.024379 | DOWN |
| 355 | CHD9 | -2.15493 | 3.58E-05 | 0.024379 | DOWN |
| 356 | HIBCH | -13.6071 | 3.88E-05 | 0.025885 | DOWN |
| 357 | RPL8 | -2.10779 | 3.98E-05 | 0.026346 | DOWN |
| 358 | PUS7L | -1.74515 | 4.52E-05 | 0.029289 | DOWN |
| 359 | RAP1B | #NAME? | 4.67E-05 | 0.03007 | DOWN |
| 360 | EIF4G3 | -3.30428 | 4.72E-05 | 0.030192 | DOWN |
| 361 | PSEN1 | -2.3199 | 4.97E-05 | 0.031594 | DOWN |
| 362 | IFT52 | #NAME? | 5.09E-05 | 0.03215 | DOWN |
| 363 | DKC1 | -3.26619 | 5.18E-05 | 0.032281 | DOWN |
| 364 | MAP3K7 | -14.589 | 5.39E-05 | 0.033168 | DOWN |
| 365 | LCOR | #NAME? | 5.47E-05 | 0.033398 | DOWN |
| 366 | TTC39C | -1.73791 | 5.59E-05 | 0.03374 | DOWN |
| 367 | SLC29A1 | -12.7874 | 6.42E-05 | 0.038498 | DOWN |
| 368 | ZNF518A | -2.54716 | 6.72E-05 | 0.039544 | DOWN |
| 369 | ING1 | -2.89449 | 6.83E-05 | 0.039708 | DOWN |
| 370 | SEH1L | -3.33463 | 6.80E-05 | 0.039708 | DOWN |
| 371 | LGALS3 | -2.07932 | 7.36E-05 | 0.04123 | DOWN |
| 372 | PPP3CA | #NAME? | 7.30E-05 | 0.04123 | DOWN |
| 373 | ATP6V1C2 | -2.01787 | 7.19E-05 | 0.04123 | DOWN |
| 374 | MBOAT2 | -4.63304 | 7.28E-05 | 0.04123 | DOWN |
| 375 | PCF11 | -1.96803 | 7.38E-05 | 0.04123 | DOWN |
| 376 | IKBKB | -5.1595 | 7.44E-05 | 0.04123 | DOWN |
| 377 | SSFA2 | #NAME? | 7.89E-05 | 0.042981 | DOWN |
| 378 | SEC31A | #NAME? | 7.94E-05 | 0.043011 | DOWN |
| 379 | EWSR1 | #NAME? | 8.13E-05 | 0.043288 | DOWN |
| 380 | CEP57L1 | #NAME? | 8.08E-05 | 0.043288 | DOWN |
| 381 | BACH2 | #NAME? | 8.09E-05 | 0.043288 | DOWN |
| 382 | NUPL2 | #NAME? | 8.37E-05 | 0.043841 | DOWN |
| 383 | TEX2 | #NAME? | 8.37E-05 | 0.043841 | DOWN |
| 384 | GGA3 | #NAME? | 8.46E-05 | 0.043854 | DOWN |
| 385 | CNOT10 | -14.9753 | 8.52E-05 | 0.043907 | DOWN |
| 386 | FOXP1 | -12.505 | 8.67E-05 | 0.044177 | DOWN |
| 387 | CD47 | #NAME? | 8.75E-05 | 0.044378 | DOWN |
| 388 | SGO1 | -13.5056 | 8.83E-05 | 0.044529 | DOWN |
| 389 | GOLGB1 | #NAME? | 9.22E-05 | 0.046 | DOWN |
| 390 | RTN4 | -13.3067 | 9.30E-05 | 0.046195 | DOWN |
| 391 | XRCC6 | #NAME? | 0.01295 | >0.05 | DOWN |
| 392 | SLC29A1 | #NAME? | 0.01615 | >0.05 | DOWN |
| 393 | ZNF518A | #NAME? | 0.0118 | >0.05 | DOWN |
| 394 | KAT6B | -11.3419 | 0.049 | >0.05 | DOWN |
| 395 | ING1 | #NAME? | 0.0122 | >0.05 | DOWN |
| 396 | SEH1L | #NAME? | 0.01635 | >0.05 | DOWN |
| 397 | LGALS3 | #NAME? | 0.0439 | >0.05 | DOWN |
| 398 | PPP3CA | -13.3758 | 0.03905 | >0.05 | DOWN |
| 399 | ATP6V1C2 | -1.56771 | 0.02815 | >0.05 | DOWN |
| 400 | VPS41 | -1.01199 | 0.0306 | >0.05 | DOWN |
| 401 | MBOAT2 | -1.02122 | 0.01585 | >0.05 | DOWN |
| 402 | STAR | -13.4053 | 0.0416 | >0.05 | DOWN |
| 403 | PCF11 | #NAME? | 0.0261 | >0.05 | DOWN |
| 404 | IKBKB | #NAME? | 0.0449 | >0.05 | DOWN |
| 405 | SSFA2 | #NAME? | 0.03285 | >0.05 | DOWN |
| 406 | 9-Sep | #NAME? | 0.04705 | >0.05 | DOWN |
| 407 | SEC31A | #NAME? | 0.0154 | >0.05 | DOWN |
| 408 | EWSR1 | #NAME? | 0.0449 | >0.05 | DOWN |
| 409 | CEP57L1 | #NAME? | 0.0197 | >0.05 | DOWN |
| 410 | BACH2 | #NAME? | 0.0206 | >0.05 | DOWN |
| 411 | TBC1D1 | #NAME? | 0.0449 | >0.05 | DOWN |
| 412 | NUPL2 | #NAME? | 0.03285 | >0.05 | DOWN |
| 413 | TEX2 | #NAME? | 0.03285 | >0.05 | DOWN |
| 414 | RASSF1 | #NAME? | 0.01635 | >0.05 | DOWN |
| 415 | GGA3 | #NAME? | 0.01295 | >0.05 | DOWN |
| 416 | CNOT10 | #NAME? | 0.03885 | >0.05 | DOWN |
| 417 | SERP2 | #NAME? | 0.01515 | >0.05 | DOWN |
| 418 | RP11-434P11.2 | #NAME? | 0.03285 | >0.05 | DOWN |
| 419 | RP1-30G7.2 | #NAME? | 0.0254 | >0.05 | DOWN |
| 420 | VPREB3 | #NAME? | 0.04215 | >0.05 | DOWN |
| 421 | DVL3 | #NAME? | 0.0441 | >0.05 | DOWN |
| 422 | RP1-266L20.2 | #NAME? | 0.0424 | >0.05 | DOWN |
| 423 | RP11-435O5.2 | #NAME? | 0.00985 | >0.05 | DOWN |
| 424 | C6orf132 | -2.36954 | 0.0428 | >0.05 | DOWN |
| 425 | RP11-109L13.1 | #NAME? | 0.01635 | >0.05 | DOWN |
| 426 | HIST2H3D | #NAME? | 0.0449 | >0.05 | DOWN |
| 427 | CTD-2550O8.7 | #NAME? | 0.0449 | >0.05 | DOWN |
| 428 | AC092415.1 | #NAME? | 0.0334 | >0.05 | DOWN |
| 429 | CTC-296K1.3 | #NAME? | 0.0449 | >0.05 | DOWN |
| 430 | RP11-277P12.9 | #NAME? | 0.0449 | >0.05 | DOWN |
| 431 | RP11-293M10.5 | #NAME? | 0.0336 | >0.05 | DOWN |
| 432 | RP11-1250I15.3 | #NAME? | 0.0449 | >0.05 | DOWN |
| 433 | CTD-2537I9.5 | #NAME? | 0.0449 | >0.05 | DOWN |
| 434 | MSH6 | #NAME? | 0.0347 | >0.05 | DOWN |
| 435 | MT1E | #NAME? | 0.00005 | >0.05 | DOWN |
| 436 | OLFM1 | -3.82635 | 0.03395 | >0.05 | DOWN |
| 437 | STARD13 | -2.67323 | 0.0029 | >0.05 | DOWN |
| 438 | BTBD17 | -3.33131 | 0.0075 | >0.05 | DOWN |
| 439 | PRRC2C | #NAME? | 0.01775 | >0.05 | DOWN |
| 440 | SRRM1 | #NAME? | 0.03935 | >0.05 | DOWN |
| 441 | COQ10A | -13.8839 | 0.02295 | >0.05 | DOWN |
| 442 | MAGEA6 | #NAME? | 0.00005 | >0.05 | DOWN |
| 443 | CTD-2311M21.2 | #NAME? | 0.0003 | >0.05 | DOWN |
| 444 | UBE2Q2P6 | -4.38124 | 0.0462 | >0.05 | DOWN |
| 445 | MT1G | -4.43589 | 0.0254 | >0.05 | DOWN |
