# Supplementary figures and images for: HSPG2 overexpression independently predicts poor survival in patients with acute myeloid leukemia
Source: Cell Death Dis. 2020 Jun 30;11(6):492. doi: 10.1038/s41419-020-2694-7 (PMC7327006; doi:10.1038/s41419-020-2694-7)

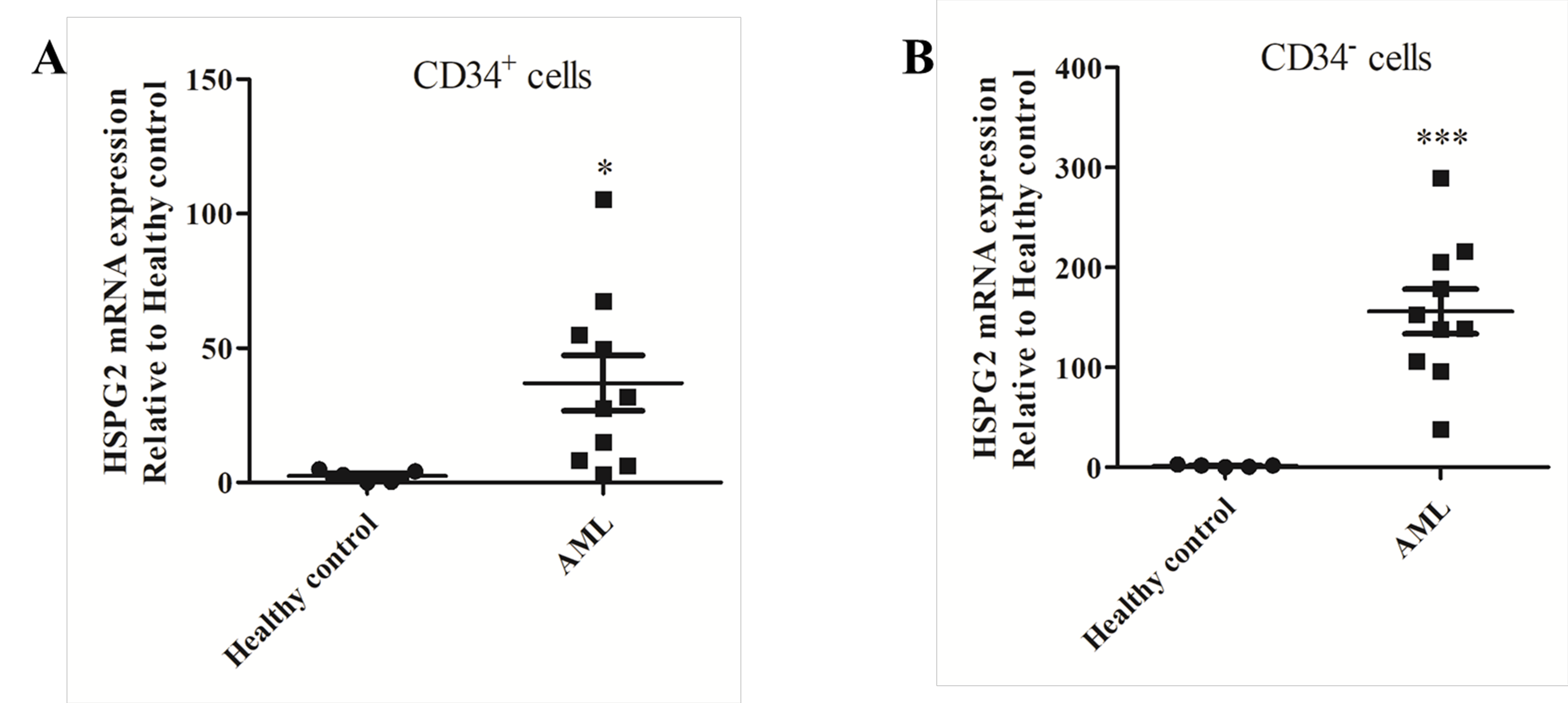

Supplement: Supplementary file 2 — Fig. S1 [file 41419_2020_2694_MOESM2_ESM.tif]
